# Design and Fabrication of High Activity Retention Al-Based Composite Powders for Mild Hydrogen Generation

**DOI:** 10.3390/ma12203328

**Published:** 2019-10-12

**Authors:** Cuiping Wang, Kairui Lin, Yuheng Liu, Xinren Chen, Hongwei Zou, Changrui Qiu, Shuiyuan Yang, Xingjun Liu

**Affiliations:** 1 College of Materials and Fujian Provincial Key Laboratory of Materials Genome, Xiamen University, Xiamen 361005, China; careylin@stu.xmu.edu.cn (K.L.); yuhengliu@hotmail.com (Y.L.); yangshuiyuan@xmu.edu.cn (S.Y.); 2 Institute of Materials Genome and Big Data, and Institute of Hydrogen and Fuel Cell, Harbin Institute of Technology, Shenzhen 518055, China; cxr1230@foxmail.com; 3 Shenzhen R&D Center for Al-based Hydrogen Hydrolysis Materials, Shenzhen 518055, China

**Keywords:** hydrogen generation, aluminum, hydrolysis reaction, gas atomization

## Abstract

Al–Bi–Sn–Cu composite powders for hydrogen generation were designed from the calculated phase diagram and prepared by the gas atomization process. The morphologies and structures of the composite powders were investigated using X-ray diffraction (XRD) and a scanning electron microscope (SEM) equipped with energy-dispersive X-ray (EDX) spectroscopy, and the results indicate that the Cu additive enhanced the phase separation between the Al-rich phase and the (Bi, Sn)-rich phase. The hydrogen generation performances were investigated by reacting the materials with distilled water. The Al–Bi–Sn–Cu powders reveal a stable hydrogen generation rate, and the Al–10Bi–7Sn–3Cu (wt%) powder exhibits the best hydrogen generation performance in 50 °C distilled water which reaches 856 mL/g in 800 min. In addition, the antioxidation properties of the powders were also studied. The Al–10Bi–7Sn–3Cu (wt%) powder has a good resistance to oxidation and moisture, which shows great potential for being the hydrogen source for fuel cell applications.

## 1. Introduction

In the era of a shortage of fossil fuels, hydrogen has been extensively regarded as a future energy source due to its cleanness, high energy, and abundance. The development and utilization of hydrogen-related technologies have received great attention in the face of the upcoming hydrogen economy [1,2,3,4,5,6]. Current hydrogen generation methods including steam reforming of ethanol [7] and water electrolysis [8] are extensively utilized to produce hydrogen to satisfy the industrial requirements. However, there are some drawbacks to these methods, such as environmental pollution, low conversion and high cost. Furthermore, these methods are not convenient for on-board hydrogen generation, which is the key issue to ensure the future hydrogen economy. Recently, hydrogen generation through the hydrolysis of metals which have a high electrochemical activity, e.g., Al [9], Mg [10], Na [11], and Zn [12], is gradually rising to realize the on-board hydrogen generation. Yavor et al. [13] studied hydrogen production by using hydrolysis on sixteen different metal powders, wherein Al, Mg and Mn powders showed high potential in being energy carriers for hydrogen production. Among them, aluminum and its alloys have received much attention. One gram of Al can generate 1.24 L H_2_ in theory, and the reaction of Al hydrolysis can be expressed as follows [14]:

2Al + 4H_2_O → 2AlOOH + 3H_2_ (1)

2Al + 6H_2_O → 2Al(OH)_3_ + 3H_2_ (2)

2Al + 3H_2_O → Al_2_O_3_ + 3H_2_ (3)

Only aluminum compounds and hydrogen are produced. In addition, the by-products AlOOH and Al(OH)_3_ can be used in fire retardant and synthetic rubber. However, it is well known that the efficiency of H_2_ generation through the hydrolysis of Al and its alloys is highly hindered by the formation of a compact inert oxidation layer [15,16]. Therefore, removing the compact inert oxidation layer to keep Al or Al alloys in an active state is essential to improve the H_2_ generation efficiency. Many efforts have been made to settle this issue. Waste Al scrap ball milling with Ni, Bi, and NaCl for 5 h were found to react completely in 0.25 M NaOH solution at 70 °C [17]. Modified γ-Al_2_O_3_ Al powders prepared by Al(OH)_3_ suspension were investigated, and 85% of the metal Al in the powder was consumed within 3 h [18]. A series of Al-Sn-In composite powders were also prepared by high energy ball milling, and they displayed high hydrolysis reactivity [19]. Furthermore, it has been reported that the Al particle size and its reaction temperature can strongly affect the hydrogen yield of aluminum hydrolysis [20]. While the ultrasonic agitation in the aluminum–water reaction can lead to a higher hydrogen generation rate and yield. Particularly, alloying Al with low melting point metals (Bi, Ga, Sn, In, etc.), has shown high efficiency to undermine the inert oxidation layer and enhance the reactivity of aluminum in the hydrolysis process [21,22,23], which offered a feasible way to facilitate hydrogen generation. Nevertheless, the additions of Ga and In greatly increased the cost. Little attention has been focused on the alloying effect of metals with higher melting points (Cu, Ca, Mn, Ni, etc.).

In our previous studies, Al-based composite powders alloying with Bi and Sn exhibited a high hydrogen generation performance due to its shell–core structure attributed to liquid phase separation [24,25]. However, the hydrogen generation rate of Al–Bi–Sn composite powders was too rapid to meet the hydrogen input condition of a hydrogen–oxygen fuel cell, resulting in the discharge of excess hydrogen [26,27]. The excessive hydrogen generation rate can be controlled by temperature and the Al/water ratio, which may not be a good method in some situations. It is necessary to study different kinds of Al-based powders that can be directly applied to different situations without extra control measures. As we all know, Cu is usually used as a cathode for the galvanic cell, and it can form the most common galvanic cell combined with Al [23]. Therefore, Cu has the potential to be an effective additive in the hydrolysis process of an Al-based alloy. In this research, Al–Bi–Sn–Cu hydrogen generation composite powders with different Cu contents were designed based on a calculated phase diagram and prepared by the gas atomization method. The morphologies and structures of the prepared powders were characterized and analyzed. Meanwhile, the hydrogen generation performances of these powders were studied in distilled water, and the reaction mechanism was proposed. Furthermore, the activity maintenance properties of the composite powders were also investigated.

## 2. Materials and Methods

### 2.1. Design of Powder Composition

**Figure 1:**
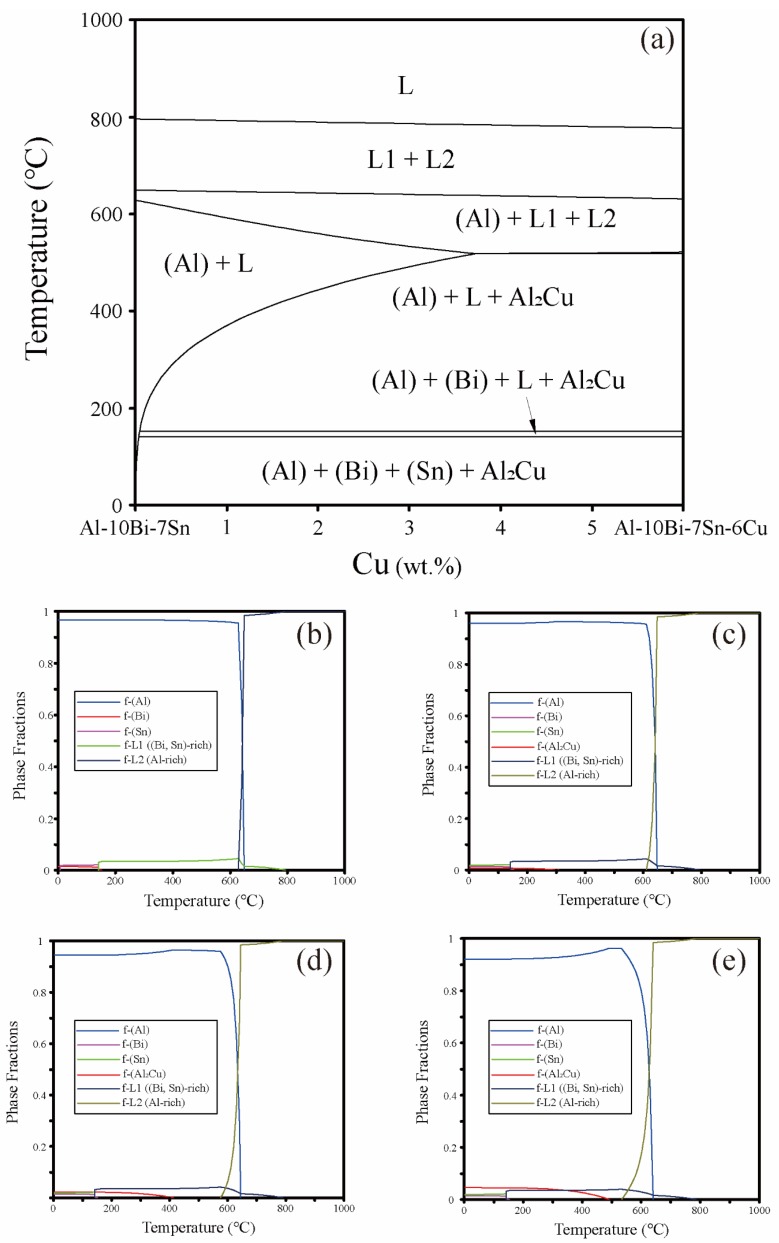
(**a**) Calculated vertical phase diagram of Al–10Bi–7Sn-(0~6)Cu wt%, and calculated phase fractions during solidification in (**b**) Al–10Bi–7Sn, (**c**) Al–10Bi–7Sn–0.5Cu, (**d**) Al–10Bi–7Sn–1.5Cu, (**e**) Al–10Bi–7Sn–3Cu alloys.

The vertical section phase diagram of Al–10Bi–7Sn-(0~6)Cu (wt%) was calculated using the thermodynamic database of Al-based alloys established by our research group, as presented in Figure 1a. From Figure 1a, we can see that there exists a stable liquid–liquid separation in this quaternary system, where the liquid phase separates into two different liquid phases, L1 + L2. The results suggest that alloys located in the compositions of liquid–liquid phase separation would form powders with two liquid phases coexisting under the rapid solidification process of gas atomization. Based on the phase diagram, we designed powders with compositions of Al–10Bi–7Sn, Al–10Bi–7Sn–0.5Cu, Al–10Bi–7Sn–1.5Cu, and Al–10Bi–7Sn–3Cu (wt%), for studying.

The calculated mole fractions of phases during solidification in Al–Bi–Sn and Al–Bi–Sn–Cu systems (Figure 1b–e) show that liquid–liquid phase separation occurred in the cooling process, then the Al-rich phase and the (Bi, Sn)-rich phase are formed. In the two phases, the mole fraction of the Al-rich phase is larger than the (Bi, Sn)-rich phase. Along with the decreasing temperature, the mole fraction of the Al-rich phase also decreases, while the mole fraction of the (Bi, Sn)-rich phase increases, which shows good agreement with the calculated phase diagram. It should be noted that the mole fraction of Al_2_Cu has significant differences in these three quaternary Al–Bi–Sn–Cu alloys, and the Al_2_Cu mole fraction in Al–10Bi–7Sn–3Cu (wt%) is much higher than that of Al–10Bi–7Sn–1.5Cu (wt%) and Al–10Bi–7Sn–0.5Cu (wt%).

### 2.2. Powder Preparation

For this research, high purity (99.9%) bulk metals of Al, Bi, Sn and Cu were taken as raw materials. The master alloys were melted in high-frequency induction smelting, then atomized using high-pressure argon gas (8 MPa). After atomization, the powders were kept in airtight chambers and then stored in an argon-atmosphere glove box for subsequent use. Four kinds of Al-based powders with 0, 0.5, 1.5, and 3 wt% Cu were prepared. For all the powders, the Bi and Sn contents were maintained unaltered for 10 wt% and 7 wt%, respectively. In this paper, the powders are marked as Al–10Bi–7Sn, Al–10Bi–7Sn–0.5Cu, Al–10Bi–7Sn–1.5Cu, and Al–10Bi–7Sn–3Cu, according to the nominal compositions of the as-atomized powders. 

### 2.3. Hydrogen Generation Measurement

**Figure 2:**
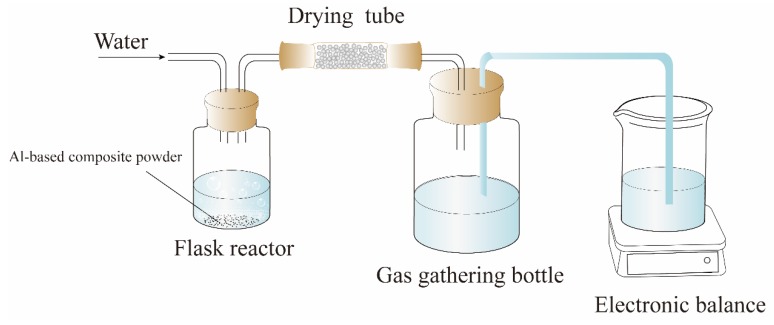
The schematic diagram of the experimental device used for hydrogen yield measurement.

The water trap method was used to measure the yield of hydrogen produced from a composite powder, and the schematic diagram of the device is shown in Figure 2. The hydrolysis processes of the powders were conducted in a 100 mL glass reactor with two outlets. In each test, the weight of the composite powders was kept at 0.3 g and placed into the glass reactor, which was performed in an argon atmosphere. Then the reactor was immersed in a water bath to keep the reaction temperature constant, and 10 mL of pre-heated distilled water (the temperature of the pre-heated water is consistent with the reaction temperature) was injected into the reactor. During the whole hydrolysis reaction, no agitation was used to promote the reaction. Generated hydrogen replaced the water in the gas gathering bottle. The hydrogen yield (mL/g) was obtained by measuring the weight of the displaced water.

### 2.4. Characterization Methods

The morphology observation and microstructure analysis of the powders was carried out by the scanning electron microscope (SEM, SU-70, Hitachi, Tokyo, Japan) equipped with an energy-dispersive X-ray (EDX) spectrometer. The X-ray diffraction pattern (XRD) of the powders was obtained by an X-ray diffractometer (D8 Advance, Bruker, Madison, WI, USA). The cross-section of the composite powders was obtained through grinding and polishing with dry emery paper, 0.25 μm polishing paste, and a velour polishing cloth. In the anti-oxidation experiments, samples were stored in a constant temperature and humidity chamber (25 °C and 60 RH%). 

## 3. Results and Discussions

### 3.1. Morphology Observation and Microstructure Analysis

**Figure 3:**
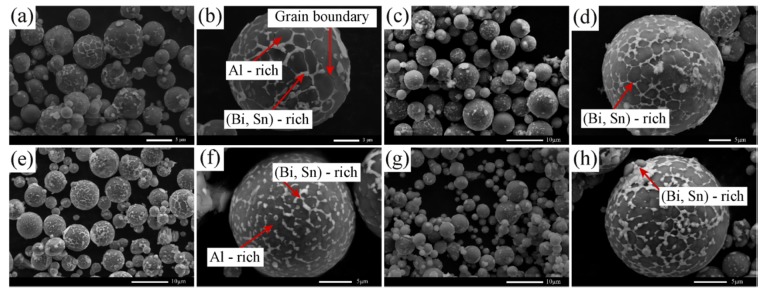
SEM images of the as-atomized Al–Bi–Sn–Cu composite powders: (**a**,**b**) Al–10Bi–7Sn, (**c**,**d**) Al–10Bi–7Sn–0.5Cu, (**e**,**f**) Al–10Bi–7Sn–1.5Cu, (**g**,**h**) Al–10Bi–7Sn–3Cu.

**Figure 4:**
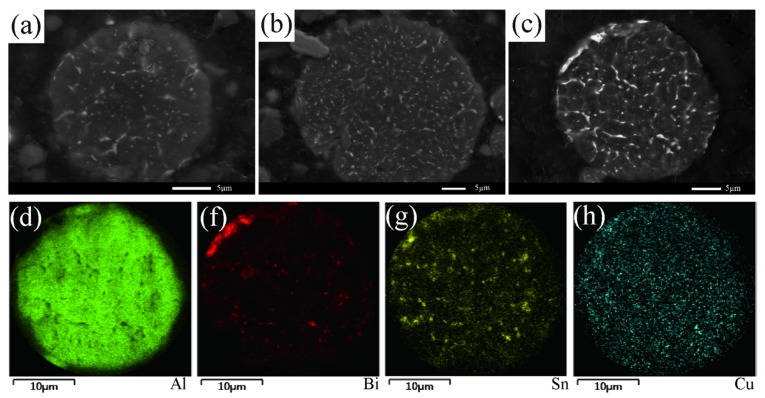
(**a**–**c**) SEM images of the cross-sections of Al–Bi–Sn–Cu powders: (**a**) Al–10Bi–7Sn–0.5Cu, (**b**) Al–10Bi–7Sn–1.5Cu, (**c**) Al–10Bi–7Sn–3Cu; (**d**–**h**) the EDS mapping of Al–10Bi–7Sn–3Cu for (**d**) Al, (**f**) Bi, (**g**) Sn, (**h**) Cu.

Figure 3 shows SEM images of the surface of as-atomized Al-based powders with different Cu contents. It can be seen from Figure 3b that the surface of Al–10Bi–7Sn alloy composite powder consisted of an Al-rich phase (gray phase) and a (Bi, Sn)-rich phase (white phase), which can be confirmed by EDX analysis. The (Bi, Sn)-rich phase was mainly aggregated on the grain boundary of the Al-rich phase in linear form, and a small part was dispersedly wrapped on the surface of the powder. It is worth noting that the ternary Al–10Bi–7Sn alloy composite powder was cracked along the grain boundary of the Al-rich phase, and numerous fissures formed on the surface. From Figure 3d, the Al–10Bi–7Sn–0.5Cu powder shows a similar morphology to that of Al–10Bi–7Sn, due to the low Cu addition. It is clear that, after the addition of Cu, the coverage area of the (Bi, Sn)-rich phase on the grain boundary of the Al-rich phase increased from partial coverage (0.5 wt% Cu) to extensive coverage (3 wt% Cu). The cross-sectional morphology of quaternary Al–Bi–Sn–Cu powders is presented in Figure 4a–c; the morphological characteristics of the cross-section were similar to the surface, whereby the (Bi, Sn)-rich phase aggregated on the grain boundary of the Al-rich phase, and the coverage area of the (Bi, Sn)-rich phase increased with the increment of Cu content. The experimental results above indicate that the Cu addition enhanced the phase separation between the Al-rich phase and the (Bi, Sn)-rich phase. The addition of Cu greatly increased the critical temperature and the interfacial tension between the Al-rich phase and the (Bi, Sn)-rich phase; the miscibility gap was enlarged as well. The reasons above made the morphologies of ternary Al-Bi-Sn powders and quaternary Al–Bi–Sn–Cu powders differ [28]. 

**Figure 5:**
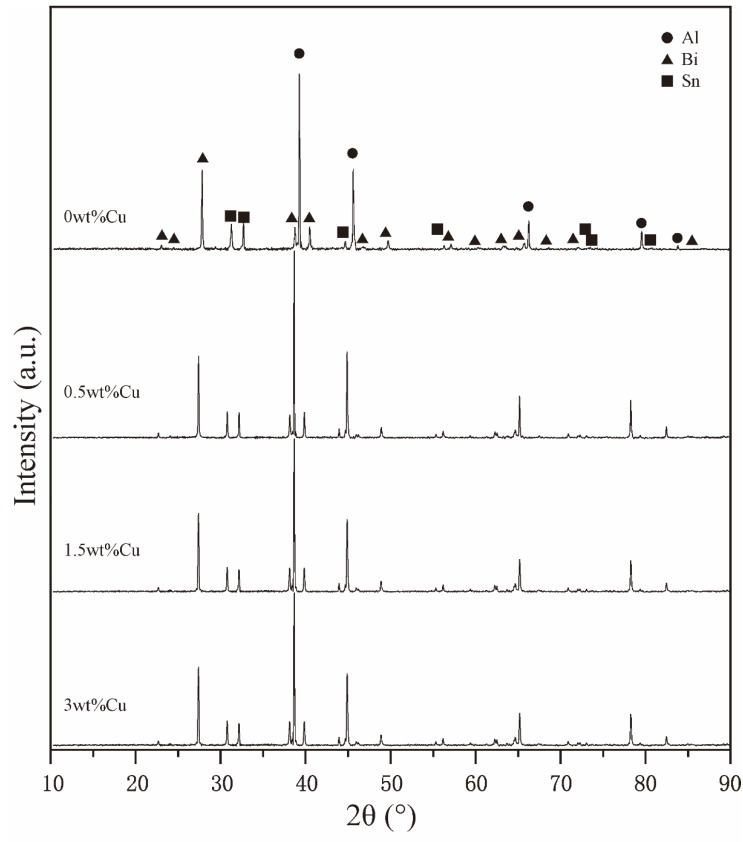
XRD patterns of the Al–10Bi–7Sn-xCu (x = 0, 0.5, 1.5, 3 wt%) composite powders.

Figure 5 presents the XRD patterns of the as-atomized composite powders. As displayed in the patterns, the characteristic diffraction peaks of Al, Bi and Sn can be obtained, implying that they did not react with each other in gas atomization, which can be attributed to the limited solid solubility and lack of compound formed in the Al–Bi–Sn system [29]. The XRD result is in good agreement with our previous research results [26]. Nevertheless, no Cu-containing phase was detected. It is noteworthy that all Al peaks were slightly shifted to the left side after the addition of Cu, therefore, we presumed that the Al (Cu) solid solution was fabricated by embedding Cu into an Al crystal lattice. Furthermore, recent work by Tian Liu et al. [30] has reported that the Al_2_Cu (θ) phase was observed in the process of solidification through TEM, and the phase fraction was too small to obtain the diffraction peak. Figure 4d–g shows the EDS element mapping of the cross-section of the Al–10Bi–7Sn–3Cu powder. Based on the above results, Cu was mainly dissolved in the Al-rich phase and also formed a small amount of the Al_2_Cu (θ) precipitated phase in the solidification, while Sn combined with Bi to form the (Bi, Sn)-rich phase.

### 3.2. Hydrogen Generation Performance

**Figure 6:**
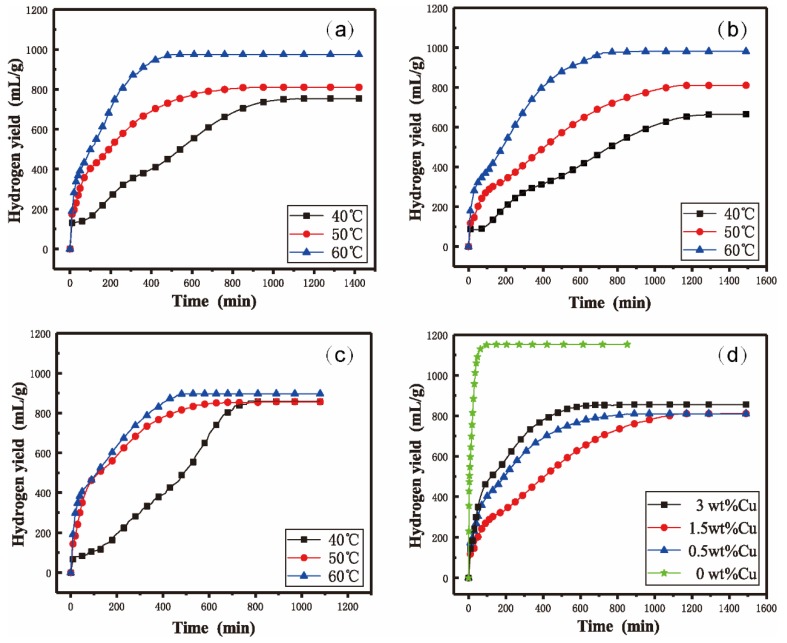
Hydrogen yield of the Al–Bi–Sn–Cu composite powders reacting with distilled water: (**a**) Al–10Bi–7Sn–0.5Cu, (**b**) Al–10Bi–7Sn–1.5Cu, (**c**) Al–10Bi–7Sn–3Cu, (**d**) Four Al–Bi–Sn–Cu powders at 50 °C.

Figure 6 shows the hydrogen yields (mL/g) of the Al–Bi–Sn–Cu composite powders’ hydrolysis with distilled water at different reaction temperatures (40 °C, 50 °C and 60 °C). As shown in Figure 6, the hydrogen generation curves of quaternary Al–Bi–Sn–Cu powders at 40 °C can be roughly divided into three stages. In stage 1, powders reacted with water upon contact resulting in the rapid hydrogen generation. This stage was the rapid raise stage of the hydrogen yield. In stage 2, the curves were almost maintained level and the hydrogen yield raised slowly. This stage was the induction time for hydrolysis. In stage 2, the Al on the surface of the Al–Bi–Sn–Cu powder had been hydrolyzed, whereas the Al inside the powder was still enfolded to prevent contact with external water, resulting in the short induction time. In stage 3, the hydrogen yield increased at a nearly constant rate until the end of the reaction. In this stage, water penetrated the interior through the cracks of the (Bi, Sn)-rich phase and restarted the hydrolysis reaction. In the reaction with 40 °C distilled water, the final hydrogen yield of Al–10Bi–7Sn–0.5Cu reached 754.18 mL/g in 1200 min, Al–10Bi–7Sn–1.5Cu reached 665.71 mL/g in 1310 min, and Al–10Bi–7Sn–3Cu reached 856.15 mL/g in 770 min. As the temperature rose to 50 °C, the number of activated molecules in the reaction increased, and the cracking of the powder was accelerated. In addition, the induction time in stage 2 disappeared at 50 °C; after the rapid hydrogen generation (stage 1), the powder was hydrolyzed to produce hydrogen at a constant rate until the reaction stopped. Compared with 40 °C, the final hydrogen yield and productive rate were both enhanced to 50 °C and reached 812.11 mL/g in 890 min for Al–10Bi–7Sn–0.5Cu, 810.75 mL/g in 1260 min for Al–10Bi–7Sn–1.5Cu, and 859.24 mL/g in 680 min for Al–10Bi–7Sn–3Cu. Likewise, the same status can be observed in the reaction temperature 60 °C. It is important to point out that the hydrogen yields of the Al–10Bi–7Sn–3Cu powder were not significantly altered by changing the reaction temperature. This may arise from the high contents of the Al_2_Cu precipitation phase, as the corrosion potential of the Al_2_Cu phase (−1.3V_SCE_) is greater than that of Al (−1.79V_SCE_), thus the electrochemical reaction between them can promote the hydrolysis process of Al [31]. Finally, the cause of the hydrogen yield of Al–10Bi–7Sn–3Cu did not show a strong correlation with reaction temperature. 

Figure 6d shows the hydrogen generation curves of Al–10Bi–7Sn-xCu (x = 0, 0.5, 1.5, 3) powders in distilled water at 50 °C. It can be seen that the trace addition of Cu effectively slowed down the hydrogen generation rate, although accompanying the decrease of the hydrogen yield. This feature allows the powders to produce hydrogen more gradually, and make it more feasible to control during the hydrogen generation process. We believe that the decrease in hydrogen yield and production rate may be due to the dissolution of Cu in the Al-rich liquid phase, which reduces the reactivity of Al, and the powders cannot react with water sharply to generate hydrogen. Additionally, under the same reaction condition (50 °C, distilled water), three kinds of Cu-containing powders have similar values in hydrogen yield, while the hydrogen production rate differs. Al–10Bi–7Sn–3Cu has the shortest reacting time, Al–10Bi–7Sn–0.5Cu the second shortest, and Al–10Bi–7Sn–1.5Cu the longest. The results did not show a clear trend and further research is necessary to determine the optimal addition of Cu. 

**Figure 7:**
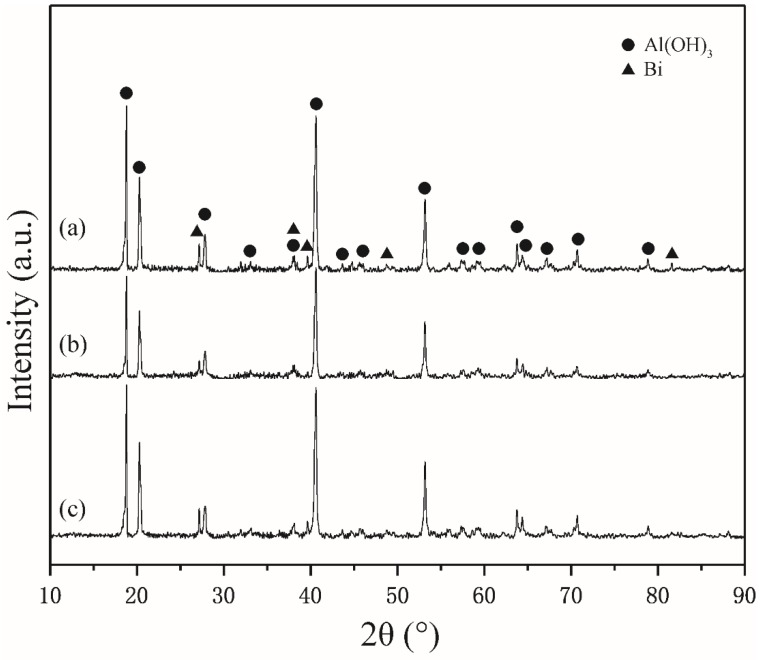
XRD patterns of Al–Bi–Sn–Cu powders hydrolysis products: (**a**) Al–10Bi–7Sn–0.5Cu, (**b**) Al–10Bi–7Sn–1.5Cu, (**c**) Al–10Bi–7Sn–3Cu.

According to the previous research, only Al was consumed during the hydrolysis of Al-Bi-Sn composite powders; Bi and Sn retain their original form in the process. Figure 7 shows the XRD pattern of the Al–Bi–Sn–Cu powders hydrolysis product, the characteristic peak of Al(OH)_3_ and Bi can be detected in the pattern, suggesting that Bi does not react with distilled water, and Al is hydrolyzed to generate hydrogen. No Sn characteristic peak was measured. It may be attributed to the low Sn contents or the dispersal and deletion of large-scale regular structures in a hydrolysis product. The reason why there was no Cu peak is the same as the as-atomized powders. Al (Cu) solid solution was formed by Cu and a part of Al, making these Al inactive and unable to be hydrolyzed. It was taken in account that the hydrogen yield of powders is lower than the theory yield. In addition, the Al_2_Cu phase acted as a cathode site to promote the hydrogen generation of the Al matrix, while not consumed by the Al_2_Cu phase itself. Thus, Cu may still remain during the forms of Al (Cu) solid solution and the Al_2_Cu phase. Bi does not react with alkali under normal conditions. However, Al(OH)_3_ is easy to react with alkali; putting the hydrolysis product into a low concentration alkali solution can dissolve the Al(OH)_3_. Therefore, the Bi could be recycled by filtering, and can be used for the preparation of the powders repeatedly. 

### 3.3. Activity Maintenance Properties

**Figure 8:**
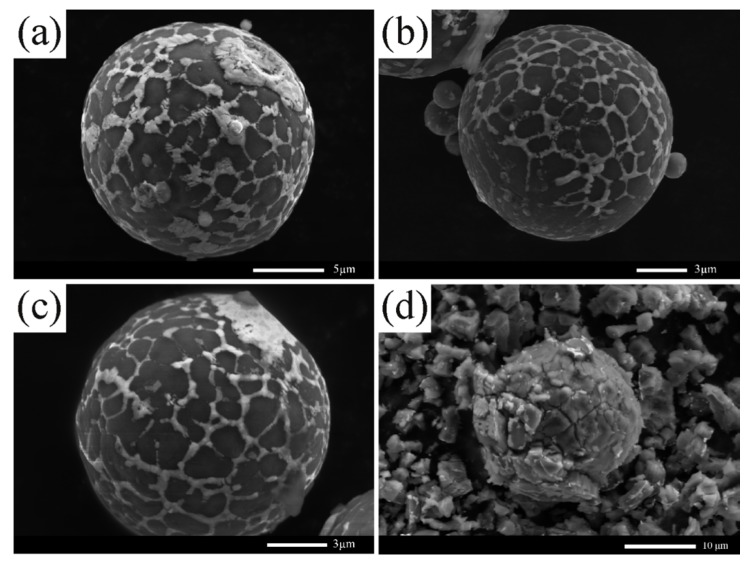
SEM images of the Al–10Bi–7Sn and Al–10Bi–7Sn–3Cu powders after being stored (25 °C and 60 RH%) for different time: (**a**) Al–10Bi–7Sn–3Cu, 6 h, (**b**) Al–10Bi–7Sn–3Cu, 12 h, (**c**) Al–10Bi–7Sn–3Cu, 24 h, (**d**) Al–10Bi–7Sn, 12h.

**Figure 9:**
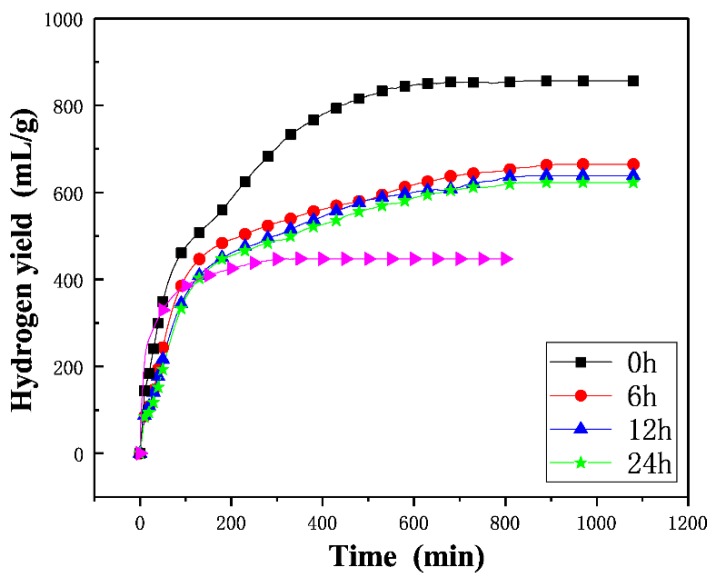
Hydrogen yields of the Al–10Bi–7Sn–3Cu composite powder reacting with 50 °C distilled water after being stored (25 °C and 60 RH%) for different times.

Traditionally, Al-based hydrogen generation materials are easy to passivate in air-exposed conditions [9], especially since the humidity of the air could accelerate the oxidation and inactivation processes. Thus, it is very important to monitor the activity of the powders when exposed to the air. In order to investigate the activity maintenance properties of the Al-based composite powders, the Al–10Bi–7Sn and Al–10Bi–7Sn–3Cu powders were stored at a constant temperature and humidity chamber (25 °C and 60 RH%) for different times. The SEM images of the powders after being aged are presented in Figure 8. It is obvious that the morphology of Al–10Bi–7Sn–3Cu powder remains almost unchanged after 6 to 24 h of aging, and the (Bi, Sn)-rich phase on the surface of the powder has a small amount of shedding (Figure 8a–c). However, after the Al–10Bi–7Sn powder was aged (Figure 8d), due to its contact with oxygen and moisture in the aging process, the powder no longer maintains the initial morphology, and the crack propagates along the grain boundary, causing the powder to become ruptured and passivated. Figure 9 shows the hydrogen generation curves of Al–10Bi–7Sn and Al–10Bi–7Sn–3Cu powders after aging for different times. The Al–10Bi–7Sn–3Cu powder has a reduced hydrogen yield (about 190 mL/g) after 6 h of aging compared with the unaged powder, which was probably due to the partial particles forming dense oxidation layers in the aging process and hindering the hydrolysis reaction. Then, we prolonged the aging time to 12 or 24 h; there was only a slight decrease in hydrogen yields, and the rate of hydrogen generation remained unchanged. It is important to point out that the hydrogen generation performance of the Al–10Bi–7Sn powder was greatly reduced because of the rupture and passivation of the powder. From the results above, a conclusion can be reached that the Al–10Bi–7Sn–3Cu powder exhibits a good activity maintenance property, providing a rather promising way for protecting the activated Al-based hydrogen generation materials and application to a proton exchange membrane fuel cell. 

## 4. Conclusions

In this work, the activated ternary Al–Bi–Sn and quaternary Al–Bi–Sn–Cu composite powders were prepared via the gas atomization method. The Cu addition in the ternary Al–Bi–Sn powder enhanced the phase separation between the Al-rich phase and the (Bi, Sn)-rich phase. The Cu mainly dissolved in a Al-rich liquid to fabricate a Al(Cu) solid solution, and partially formed the Al_2_Cu (θ) precipitated phase in the solidification. The hydrogen generation performance in distilled water was investigated, and the addition of Cu made the hydrogen generate more gradually, which can be adapted to realize the hydrogen generation in a more controlled way. The Al–10Bi–7Sn–3Cu powder showed the best performance among all the prepared quaternary Al–Bi–Sn–Cu powders when the reaction temperature was below 50 °C. The reaction temperature did not have an obvious effect on the hydrolysis of the Al–Bi–Sn–3Cu powder, whereas a strong correlation was shown in the Al–10Bi–7Sn–0.5Cu and Al–10Bi–7Sn–1.5Cu powders. Furthermore, the Al–10Bi–7Sn–3Cu powder also showed a good resistance to oxidation and moisture, thus providing a new method to protect hydrogen-producing materials. Further work will be continued on determining the optimal addition of Cu, and exploring the possibility of using such a composite powder in proton exchange, membrane proton exchange and membrane fuel cell applications. 

## References

[1] Fan M.Q., Xu F., Sun L.X. (2007). Studies on hydrogen generation characteristics of hydrolysis of the ball milling Al-based materials in pure water. Int. J. Hydrogen Energy.

[2] Soler L., Macanas J., Munoz M., Casado J. (2007). Aluminum and aluminum alloys as sources of hydrogen for fuel cell applications. J. Power Sources.

[3] Nizovskii A.I., Belkova S.V., Novikov A.A., Trenikhin M.V., Myshlyavtsev A.V., Likholobov V.A., Yusha V.L. (2015). Hydrogen production for fuel cells in reaction of activated aluminum with water. Oil and Gas Engineering.

[4] Budak Y., Devrim Y. (2019). Comparative study of PV/PEM fuel cell hybrid energy system based on methanol and water electrolysis. Energy Convers. Manag..

[5] Neef H.J. (2009). International overview of hydrogen and fuel cell research. Energy.

[6] Chen Y.H., Chen C.Y., Lee S.C. (2011). Technology forecasting and patent strategy of hydrogen energy and fuel cell technologies. Int. J. Hydrogen Energy.

[7] Sharma Y.C., Kumar A., Prasad R., Upadhyay S.N. (2017). Ethanol steam reforming for hydrogen production: Latest and effective catalyst modification strategies to minimize carbonaceous deactivation. Renew. Sustain. Energy Rev..

[8] Li A., Sun Y., Yao T., Han H. (2018). Earth-Abundant Transition-Metal-Based Electrocatalysts for Water Electrolysis to Produce Renewable Hydrogen. Chem. Eur. J..

[9] Wang H.Z., Leung D.Y.C., Leung M.K.H., Ni M. (2009). A review on hydrogen production using aluminum and aluminum alloys. Renew. Sustain. Energy Rev..

[10] Tan Z., Ouyang L., Liu J., Wang H., Shao H., Zhu M. (2018). Hydrogen generation by hydrolysis of Mg-Mg_2_Si composite and enhanced kinetics performance from introducing of MgCl_2_ and Si. Int. J. Hydrogen Energy.

[11] Herzog F., Glaubitz D. (1990). Production of Hydrogen from the Na/Nah-Process. Int. J. Hydrogen Energy.

[12] Wegner K., Ly H.C., Weiss R.J., Pratsinis S.E., Steinfeld A. (2006). In situ formation and hydrolysis of Zn nanoparticles for H_2_ production by the 2-step ZnO/Zn water-splitting thermochemical cycle. Int. J. Hydrogen Energy.

[13] Yavor Y., Goroshin S., Bergthorson J.M., Frost D.L. (2015). Comparative reactivity of industrial metal powders with water for hydrogen production. Int. J. Hydrogen Energy.

[14] John P., George T. (2008). Reaction of Aluminum with Water to Produce Hydrogen. US Dep. Energy.

[15] Parmuzina A.V., Kravchenko O.V. (2008). Activation of aluminium metal to evolve hydrogen from water. Int. J. Hydrogen Energy.

[16] Czech E., Troczynski T. (2010). Hydrogen generation through massive corrosion of deformed aluminum in water. Int. J. Hydrogen Energy.

[17] Ho C.Y., Huang C.H. (2016). Enhancement of hydrogen generation using waste aluminum cans hydrolysis in low alkaline de-ionized water. Int. J. Hydrogen Energy.

[18] Fang C.S., Gai W.Z., Deng Z.Y. (2014). Al Surface Modification by a Facile Route. J. Am. Ceram. Soc..

[19] Du Preez S.P., Bessarabov D.G. (2018). Hydrogen generation by the hydrolysis of mechanochemically activated aluminum-tin-indium composites in pure water. Int. J. Hydrogen Energy.

[20] Yavor Y., Goroshin S., Bergthorson J.M., Frost D.L., Stowe R., Ringuette S. (2013). Enhanced hydrogen generation from aluminum-water reactions. Int. J. Hydrogen Energy.

[21] Fan M.Q., Sun L.X., Xu F. (2011). Hydrogen production for micro-fuel-cell from activated Al-Sn-Zn-X (X: hydride or halide) mixture in water. Renew. Energy.

[22] Huang T., Gao Q., Liu D., Xu S., Guo C., Zou J., Wei C. (2015). Preparation of Al-Ga-In-Sn-Bi quinary alloy and its hydrogen production via water splitting. Int. J. Hydrogen Energy.

[23] Wei C., Liu D., Xu S., Cui T., An Q., Liu Z., Gao Q. (2018). Effects of Cu additives on the hydrogen generation performance of Al-rich alloys. J. Alloys Compd..

[24] Wang C., Liu Y., Liu H., Yang T., Chen X., Yang S., Liu X. (2015). A Novel Self-Assembling Al-based Composite Powder with High Hydrogen Generation Efficiency. Sci. Rep..

[25] Liu Y., Liu X., Chen X., Yang S., Wang C. (2017). Hydrogen generation from hydrolysis of activated Al-Bi, Al-Sn powders prepared by gas atomization method. Int. J. Hydrogen Energy.

[26] Pukrushpan J.T., Stefanopoulou A.G., Peng H. (2004). Control of fuel cell breathing. IEEE Control Syst. Mag..

[27] Edwards P.P., Kuznetsov V.L., David W.I.F., Brandon N.P. (2008). Hydrogen and fuel cells: Towards a sustainable energy future. Energy Policy.

[28] Kaban I., Hoyer W. (2008). Effect of Cu and Sn on liquid-liquid interfacial energy in ternary and quaternary Al-Bi-based monotectic alloys. Mater. Sci. Eng. A.

[29] Al-Bi-Sn Liquidus Projection of Ternary Phase Diagram. https://materials.springer.com/isp/phase-diagram/docs/c_0950975.

[30] Liu T., Leazer J.D., Menon S.K., Brewer L.N. (2018). Microstructural analysis of gas atomized Al-Cu alloy feedstock powders for cold spray deposition. Surf. Coat. Technol..

[31] Kim M., Eom K., Kwon J., Cho E., Kwon H. (2012). On-board hydrogen production by hydrolysis from designed Al-Cu alloys and the application of this technology to polymer electrolyte membrane fuel cells. J. Power Sources.

